# Editorial: Autism spectrum disorders and metal dyshomeostasis, volume II

**DOI:** 10.3389/fnmol.2023.1172769

**Published:** 2023-03-14

**Authors:** Mukesh K. Pandey, Andreas M. Grabrucker, Sunil Q. Mehta

**Affiliations:** ^1^Department of Radiology, Mayo Clinic, Rochester, MN, United States; ^2^Department of Biological Sciences, University of Limerick, Limerick, Ireland; ^3^Health Research Institute, University of Limerick, Limerick, Ireland; ^4^Bernal Institute, University of Limerick, Limerick, Ireland; ^5^Department of Pediatric and Adolescent Medicine, Mayo Clinic, Rochester, MN, United States; ^6^Department of Psychiatry and Psychology, Mayo Clinic, Rochester, MN, United States

**Keywords:** autism, biometals, isotopic compositions, γ-aminobutyric, valproic acid, selenoproteins

The success of finding a cure for any disease depends on the fundamental understanding of disease pathology, causative factor(s), and interventional feasibility. Autism spectrum disorder (ASD) is no different, but even though ASD has been known for a long time, our basic understanding of causative factor(s) and the biochemical basis of disease pathology is extremely limited. Based on the latest report published by the Centers for Disease Control and Prevention (CDC) USA, 1 in every 44 children is diagnosed with ASD.^1^ This is a three-fold increase in ASD prevalence in the last 18 years. The caring cost for all American ASD population was $268 billion in 2015 and is expected to rise to $461 billion by 2025.^2^ It is a growing concern, and our efforts must be expedited to better understand the disease pathology and causative factor(s). So far, it is clear that ASD is a neurodevelopmental disorder with a complex etiology and could have either environmental or genetic risk factors or a combination of both. To broaden our understanding of non-genetic factors, in this Research Topic of “*Autism Spectrum Disorders and Metal Dyshomeostasis, Volume II*,” we focused on covering the role of biometals and their isotopic compositions, γ-aminobutyric acid, valproic acid, and selenoproteins on ASD pathogenesis.

One of the articles included in this Research Topic by Zang et al. studied the role of valproic acid (VPA) exposure on neuronal development during pregnancy as a potential risk factor for ASD. Previously, the biometal zinc was used as a prenatal treatment to prevent VPA-induced impairments in a rat model of autism (Cezar et al., 2018). In this study, authors have used human dorsal forebrain organoids, exposed them to variable doses of VPA, and found significant defects in neurogenesis. Furthermore, authors have supported their findings with morphological analysis, RNA sequencing, and reported effects on critical pathways, including activation of Wnt/β-catenin and transcriptomic analysis. Additionally, this study shows the application of cerebral organoid model as a tool to study the effect of other environmental toxins on neuronal development.

Miller et al. investigated the connection between the isotopic composition of serum zinc and copper in healthy and ASD children. This is a one-of-a-kind study, where authors have looked not only at a particular biometal and its involvement in ASD but also focused on its isotopic composition and its relevance in ASD pathology. Although the authors reported no significant difference in the isotopic composition of serum zinc or serum copper in age-matched ASD vs. healthy controls, they found enrichment of serum copper as ^65^Cu in boys with respect to healthy adults in comparison with previously reported isotopic composition data.

In another study, the team at Mayo Clinic studying non-genetic factors associated with ASD had previously reported a significantly lower level of serum Se (116.83 ± 14.84 ng/mL) in ASD boys compared to healthy boys (128.21 ± 9.11 ng/mL; *p* < 0.005) (Mehta et al.), now investigated the role of selenoproteins in neurodevelopment and neurological function in association with ASD as a review article (Behl et al.). Since most Se (>80%) is associated with selenoproteins, it is prudent to summarize previous studies connecting the role of selenoproteins, Se, and ASD. Given lower levels of Se in ASD children, it is critical to understand the reasons for a lower level of serum Se in ASD and their downstream effect on neuronal development. The authors compiled a complete list of all 25 known selenoproteins, their role in brain development and function, along with possible ways of Se supplementation. In addition, they pointed out that the primary function of selenoproteins is to manage oxidative stress. In a situation of lower Se, selenoproteins might not function effectively, increasing oxidative stress that may damage the developing brain. However, more studies must be conducted to better understand various non-genetic factors and their association with ASD.

Choi et al. studied inhibitory signaling in ASD. Synaptic and extrasynaptic GABA type A receptors (GABA_A_Rs) control neuronal inhibition. The unique functions of synaptic and extrasynaptic GABA_A_R subtypes in inhibition depend on the subunit composition. The authors extracted endogenous GABA_A_Rs with subunits α1 and α4 from adult murine forebrains and analyzed their subunit composition in the present study. They discovered that the α1 and α4 subunits engage with different sets of binding proteins to produce discrete populations of GABA_A_Rs. Moreover, they found that the two receptor subtypes varied in the amount of phosphorylation in the α3 subunit, which co-purifies with the α1 and α4 subunits. Mutation of serine phosphorylation sites causes the production of a novel GABA_A_R that contains an α1 and α4 subunit and shows increased expression on the plasma membrane. According to these results, phosphorylation of serine S408 and 409 is crucial in defining the subtype-specific assembly of GABA_A_Rs and, consequently, the effectiveness of neuronal inhibition (Choi et al.).

In summary, this Research Topic presents studies focused on understanding the role of various non-genetic factors, including biometals and their isotopic composition, γ-aminobutyric acid, valproic acid, and selenoproteins in neuronal development related to ASD. The role of Se and selenoproteins in ASD is an emerging target to focus on future research studies to better understand its impact on ASD origin and pathophysiology, along with the translation of research findings from the other studies included in this Research Topic.

## Author contributions

All authors listed have made a substantial, direct, and intellectual contribution to the work and approved it for publication.

## Footnotes

^1^ https://www.cdc.gov/ncbddd/autism/data.html

^2^ https://www.autismspeaks.org/science-news/autism-and-health-special-report-autism-speaks

## References

[B1] Cezar LC Kirsten TB da Fonseca CCN de Lima APN Bernardi MM Felicio LF. Zinc as a therapy in a rat model of autism prenatally induced by valproic acid. Prog Neuropsychopharmacol Biol Psychiatry (2018) 84 (Pt A):173–80. 10.1016/j.pnpbp.2018.02.008. 29481896

